# Navigating change: the role of change management strategies and cultural factors in Saudi Arabian organizations

**DOI:** 10.3389/fpsyg.2025.1551902

**Published:** 2025-09-01

**Authors:** Chiraz Mohamed Rouissi

**Affiliations:** Department of Human Resources Management, College of Business at Alkamil, University of Jeddah, Jeddah, Saudi Arabia

**Keywords:** change management, cultural factors, organizational change, employee engagement, Saudi Arabia, culture

## Abstract

**Background:**

Organizational change remains a critical challenge in the industrial sectors of the Kingdom of Saudi Arabia, where leadership practices and deep-rooted cultural norms significantly influence transformation efforts. Despite a growing body of global change management literature, limited empirical research addresses the interplay between cultural dynamics and change management within the Saudi context.

**Objective:**

This study examines how change management techniques and cultural elements jointly affect employee engagement and performance in Saudi Arabia’s industrial sector. Kurt Lewin’s change management model serves as the theoretical foundation, providing a structured lens for analyzing transformation processes.

**Methods:**

A quantitative research design was adopted, using a self-administered survey completed by 477 employees from diverse industrial firms across Saudi Arabia. A pilot study ensured the instrument’s validity and reliability. Structural Equation Modeling (SEM) was employed to evaluate relationships among change management practices, cultural factors, and employee outcomes.

**Results:**

The findings reveal that leadership—when aligned with culturally appropriate practices—significantly enhances employee engagement and performance. Transparent communication, employee participation, and targeted training were identified as critical enablers of effective change. Furthermore, cultural factors moderated the impact of change management strategies, emphasizing the necessity of context-sensitive approaches.

**Conclusion:**

This study contributes theoretically by integrating cultural considerations into change management frameworks, addressing a gap in region-specific organizational research. Practically, the results offer actionable insights for leaders and HR professionals aiming to design culturally responsive change strategies. The novelty of this research lies in its empirical focus on the Saudi industrial sector, providing evidence-based guidance for managing change in culturally complex environments.

## 1 Introduction

In the fast-evolving global marketplace, organizations are compelled to adapt swiftly to the dynamic landscape shaped by technological advancements, regulatory shifts, and societal changes (Mintzberg, 1994; Radu, 2023). This imperative for transformation is particularly pronounced in Saudi Arabia, where the industrial sector is undergoing a profound metamorphosis catalyzed by a confluence of internal reforms and external influences (Gashgari, 2017). Notably, the ambitious Vision 2030 initiative exemplifies this drive for change by aiming to diversify the economy and reduce dependence on oil (Saudi Vision 2030, 2016; AL-Malaise et al., 2022). Effective change management emerges as a cornerstone practice, fostering sustainable growth and innovation while aligning organizational trajectories with national ambitions, and charting a path toward a promising future amid rapid upheavals (Hayes, 2018; Scott Jones, 2019).

However, implementing organizational change in Saudi Arabia is particularly challenging due to cultural rigidity, hierarchical decision-making structures, and workforce dynamics deeply rooted in traditional and religious values (Polyakov, n.d.; Trist and Murray, 1990). Resistance to change, therefore, becomes a recurrent theme (Oreg, 2006; Lewin, 1948), often exacerbated by distrust, poor communication, and the absence of employee involvement (Ford et al., 2008; Kotter, 2012). This highlights a need for a culturally responsive approach that integrates leadership, communication, and training as core mechanisms to enhance employee engagement and change acceptance (Lewin, 1951; Mauss et al., 2023).

Resistance to change, therefore, becomes a recurrent theme (Oreg, 2006), often exacerbated by distrust, poor communication, and the absence of employee involvement (Ford et al., 2008). This highlights a need for a culturally responsive approach that integrates leadership, communication, and training as core mechanisms to enhance employee engagement and change acceptance.

While some local studies have explored change practices in specific contexts—such as healthcare (Alharbi, 2018) or knowledge management (Alsereihy and El Emary, 2012)—the majority are descriptive rather than analytical, lacking a coherent theoretical framework to unify findings or assess interactions between change practices, culture, and employee outcomes (Nunnally and Bernstein, 1994). This study responds to that gap.

Kotter and Schlesinger (1979) accentuate the multifaceted methodologies pivotal in managing resistance to change, which encompass educational initiatives, participatory approaches, effective communication strategies, supportive frameworks, negotiation tactics, manipulative techniques, and coercive measures. Organizational resistance to change often stems from factors such as distrust, past triumphs, and resource allocation dilemmas, underscoring the criticality of adept change management practices in fostering innovation (Buchanan and Addison, 1998; Kotter, 2012).

Given the complexity of change in culturally conservative and economically developing regions, this study seeks to answer:

*To what extent do change management practices and cultural factors influence employee engagement and performance during organizational transformation in Saudi Arabia’s industrial sector?*

Contrary to a purely exploratory or qualitative approach, this study uses a quantitative design to empirically test the relationships among key variables and to validate a conceptual model using Structural Equation Modeling (SEM).

The structure of this research unfolds as follows: Section 1 provides the introduction; Section 2 presents a literature review on change management and cultural influences on organizational performance; Section 3 outlines the methodology; Section 4 reports the findings; and Section 5 offers a discussion and practical implications for organizational change in Saudi Arabia.

## 2 Literature review

Change management refers to the structured transition of individuals, teams, and organizations from a current state to a desired future one (Hiatt, 2006). Central to this process is employee engagement, a psychological state reflecting involvement, enthusiasm, and dedication to organizational goals (Kahn, 1990; Schaufeli et al., 2002). High engagement has been linked to improved performance, better adaptability, and reduced resistance during change.

However, organizational culture—defined as shared values, norms, and behavioral expectations (Schein, 2010)—can either facilitate or hinder engagement. In Saudi Arabia, cultural factors such as high power distance, collectivism, and uncertainty avoidance may delay or obstruct change unless the approach is carefully adapted (Hofstede, 2001; House et al., 2004). Despite recognition of these cultural influences, there remains a paucity of empirical studies examining how culture moderates the relationship between change practices and outcomes in Saudi industrial organizations.

Several studies have addressed facets of change in the region. For instance, Akbar et al. (2019, 2020) and Keshta et al. (2017) examined software firms implementing structured models like CMMI, while Alharbi (2018) emphasized transformational leadership in healthcare. However, these studies often isolate one factor (e.g., leadership) without accounting for how multiple change levers—such as participation, communication, and training—interact with cultural constraints.

For instance, Aboyassin and Sultan (2021) highlight the role of leadership adaptability in Middle Eastern change initiatives, while Alhawari et al. (2022) examine how digital readiness mediates employee engagement during organizational transitions in the Gulf region.

Radu et al. (2023) further underscore the importance of culture-informed leadership and trust-building in enhancing change acceptance. These contemporary perspectives expand upon classic models by situating change within modern digital, cultural, and geopolitical realities, especially in contexts like Saudi Arabia undergoing rapid transformation under Vision 2030.

Kebe et al. (2025) demonstrate that leadership behaviors aligned with local cultural values significantly influence employee engagement and change receptivity in developing economies. Their study highlights how cultural congruence in leadership—especially communication that honors hierarchy and collectivism—enhances trust and change success. Likewise, Alotaibi (2025) reveals how participative leadership fosters digital transformation by building an organizational culture supportive of innovation and identity formation. Together, these studies suggest that leadership must be culturally attuned and participatory to effectively navigate digital-era change in Saudi Arabia.

Moreover, research is divided on the root causes of resistance. While some scholars attribute resistance to entrenched cultural attitudes (Al-Haddad and Kotnour, 2015), others (Ford et al., 2008) argue that miscommunication and lack of transparency are the dominant drivers. These conflicting perspectives underscore the need for a multi-variable, integrative model that examines how various change management strategies work within specific cultural environments.

To analyze these complex interactions, this study adopts Lewin’s (1947) three-stage change model: Unfreeze → Change → Refreeze. While more contemporary frameworks such as ADKAR or agile models emphasize rapid, decentralized change, they assume a flexible, empowered organizational culture—which does not reflect the reality of many Saudi industrial firms. These firms often operate in top-down, hierarchical structures, where cultural values prioritize stability, deference to authority, and cautious transformation.

Lewin’s model is better suited to such contexts for several reasons: First, it provides a psychologically grounded, step-by-step process, allowing for incremental adaptation—an essential feature in rigid environments. Second, it emphasizes behavioral change and social re-norming, rather than only technical or procedural shifts. Third, it has been successfully applied in both traditional and modern sectors (Burnes, 2004; Cummings et al., 2016) and remains relevant in culturally conservative environments like Saudi Arabia.

**Figure 1:**
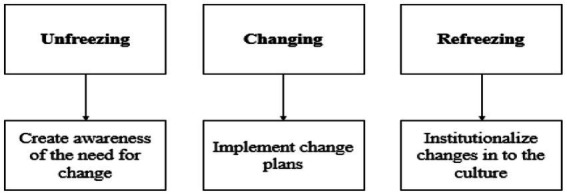
Kurt Lewin change model (Lewin, 1947).

Thus, Lewin’s model is not selected for simplicity alone, but for its cultural congruence, strategic logic, and empirical robustness in structured, hierarchical systems (see Figure 1; Table 1).

**Table 1:** Change management model (source: authors’ elaboration).

Stage	Challenges	Solutions
Unfreezing	Lack of awareness and Resistance to change	Implement the knowledge management strategies
		Provide leadership skills development programs
		Validate resistance to change scale
		Address structural challenges and lack of empowerment
Changing	Companies in Saudia facing challenges	Develop workflow models for KM and RCM implementation
		Integrate LSS with Kotter’s model for healthcare services
		Implement the CMMI Level 2 practices in software development
		Develop change management model for circular economy
		Address challenges in GSD with agile requirements management
		Utilizing the AI and ABV for public sector transformation
Refreezing	Lack of adaption	Encourage continuous improvement and knowledge sharing
		Develop long-term strategies for sustainable urbanization
		Align organizational culture with the change management goals
		Promote women’s leadership roles and address the cultural barriers
		Align technology management with environmental goals

Despite the widespread use of change models globally, few studies have explored how culturally rooted factors moderate the success of change management practices in Saudi Arabia’s industrial sector. This study addresses that gap by integrating cultural context into a quantitative, model-driven investigation, using Structural Equation Modeling (SEM) to assess the relationships among communication, leadership, participation, training, culture, employee engagement, and performance.

### 2.1 Hypothesis

For this research, data were meticulously collected from employees of industrial companies operating in the Kingdom of Saudi Arabia.

These findings collectively support the hypothesis that heightened to verify the impact of the practice of change management in the Saudi context. The thoroughness of this study is evident in the attention given to the employees’ engagement in the industrial companies and their cultural factors, ensuring a comprehensive understanding of the subject. Accordingly, we hypothesize the following:

*Hypothesis 1:* States that communication *concerns positively* on Employee Engagement and Performance.

*The first sub-hypothesis (H1.1):* Employee participation in decision-making *has a positive effect* on Employee Engagement and Performance.

*The second sub-hypothesis (H1.2):* Training has a positive effect on Employee Engagement and Performance.

*The third sub-hypothesis (H1.3):* Leadership positively affects Employee Engagement and Performance.

*Hypothesis (H2):* The culture of Saudi Arabia positively impacts Employee Engagement and Performance.

These hypotheses offer an empirically testable framework for understanding how change efforts interact with deeply rooted cultural dynamics, and what mechanisms drive employee acceptance and performance during transformation efforts in the Saudi context.

## 3 Materials and methods

This research uses a quantitative approach to investigate the role of change management strategies and cultural factors in Saudi Arabian organizations. The quantitative method was chosen because it provides reliable, numerical data that can be analyzed objectively, allowing conclusions about the effectiveness of change management practices and their correlation with organizational culture and employee engagement.

### 3.1 The conceptual model

**Figure 2:**
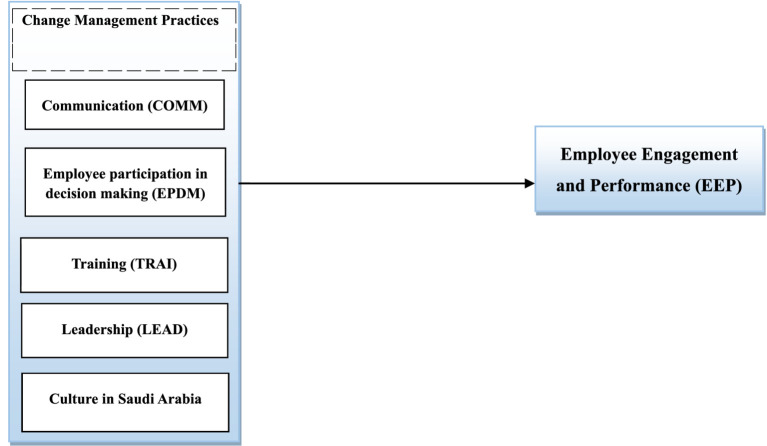
Conceptual framework (source: author’ elaboration).

The conceptual framework is mapped out as follows to validate the findings from the literature review and better understand the study context. The theoretical model is presented in Figure 2.

### 3.2 Research design

A descriptive correlational research design examined the relationships between change management strategies, cultural factors, and employee attitudes toward change. The design allows for the measurement of the impact of specific variables (such as change management practices and cultural influences) on organizational performance while also accounting for external factors that may affect the outcomes of change processes.

We proceeded by determining the parent population, which consists of Saudi industrial companies that have the characteristics for the study. Respondents are executives, project managers, finance analysts, accounting personnel, and leaders from Aramco Company, Saudi European Petrochemical Company, Arabian Petrochemical Company, ABIC Investment and Local Content Development Company (“NUSANED”), SABIC Industrial Investments Company (“SIIC”), Saudi Kayan Petrochemical Company (“SAUDI KAYAN”), and SABIC Innovative Plastics Aus GmbH Aramco Oil Pipelines Company.

The counting of questionnaire data obtained was realized through SPSS Version 2024 and Amos. This software allows describing the data and performing all desired analyses, including flat sorting and multivariate methods (see Table 2).

**Table 2:** Respondent profile.

Category		Percentage
Age of respondents	20–25 years	10.3%
	26–35 years	41.3%
	36–45 years	30.4%
	46–55 years	15.3%
	56–60 years	2.7%
Gender of respondents	Male	65%
	Female	35%
Work experience	Less than 2 years	14.9%
	2–5 years	15.1%
	6–10 years	13.6%
	10–15 years	21.6%
	16–20 years	11.1%
	Plus de 20 years	23.7%
Education level	High school diploma	3.6%
	Bachelor’s degree	25.2%
	Master’s academic	9%
	Master’s degree professionals	47.4%
	Engineering degree	11.9%
	Doctorate (PhD)	2.9%
Role	Director	20.1%
	Senior manager	49.9%
	Mid-level manager	19.7%
	Employee	10.3%

Most interviewees (41.3%) were aged 26–35 years, followed by 36–45 years (30.4%), 46–55 years (15.3%), 20–25 years (10.3%), and 56–60 years (2.7%). The age of respondents plays a critical role in response reliability, as more experienced individuals provide more accurate insights into our research context.

Most respondents (65%) were male, while only 35% were female. This gender distribution highlights the influence of respondent gender on response homogeneity, particularly in understanding the study’s focus: *“the impact of change management practices and Saudi culture on employee engagement and performance”*.

### 3.3 Sample description

For our research, we conducted basic data filtering to determine the characteristics of the sample units, including the companies and the respondents’ profiles (age, gender, work experience, education level, job role, etc.).

To determine the sample size, we followed the recommendations of Roussel et al. (2002), which specify that the sample size must meet certain criteria. These include the number of items and latent variables in the theoretical model, as well as the number of covariances in the data matrix. Additionally, the sample size depends on the estimation method used for the theoretical model.

The minimum sample size must exceed the number of covariances in the data matrix, while the maximum size aligns with a ratio of ten observations per estimated parameter. Furthermore, the use of the maximum likelihood method in confirmatory factor analyses (CFA) necessitates medium-sized samples. Consequently, we selected a convenience sample of 477 companies in Saudi Arabia, spanning diverse sectors and varying company sizes.

Regarding the characteristics of our sample, we opted for a convenience sample of individuals from diverse age groups and genders. This diversity across criteria ensures homogeneous responses. Additionally, we ensured a relatively balanced proportion of female and male *Saudi respondents*.

## 4 Data analysis results

### 4.1 Scale of exploratory factor analysis of employee engagement and performance, the culture, and the change management

To assess the factorability of the data and determine the exploratory reliability of the change management practices measurement scale, we conducted an exploratory factor analysis (EFA) using principal component analysis (PCA).

The first PCA with varimax rotation yielded satisfactory factor contributions, factor loadings, and reliability metrics (see Appendix 1). The results of the EFA for the change management practices scale revealed: KMO Measure (Kaiser-Meyer-Olkin) = 0.87 and Bartlett’s Test (*p* = 0.000), confirming the suitability of the data for factor analysis.

An Exploratory Factor Analysis (EFA) was conducted on the Employee Engagement and Performance scale using Principal Component Analysis (PCA) with varimax rotation. The results indicate a unidimensional factor structure for the scale, comprising a single variable, *“Employee Engagement and Performance,”* with 4 items.

The examination of factor loadings and factor contributions revealed satisfactory values (all above 0.5). Additionally, the Kaiser-Meyer-Olkin (KMO) measure and Bartlett’s Sphericity test yielded acceptable values. Finally, the Cronbach’s alpha for this variable demonstrated good internal consistency at the exploratory level. In addition to the exploratory factor analysis, we conducted a confirmatory factor analysis on the culture scale. The results of a first CFA show that the Kurtosis and Skewness indices are within normal ranges, while the Mardia index, which is approximately 14.997, significantly exceeds the minimum threshold set at 3.

The culture measurement model shows good fit indices. Indeed, the standardized Chi-square does not exceed the threshold of 5 set by Marsh and Hocevar (1985). The fit indices are satisfactory with a decrease in the value of the standardized Chi-square as shown in the Table 3.

**Table 3:** Exploratory factor analysis results for variables scales.

Variables	Items		Factor loadings	Factor contributions
				Engagement et rendement des employés
Employee Engagement and Performance	EEP1	Our organization is flexible and reacts quickly to changes.	0.689	0.830
	EEP2	Organizational goals provide clear guidelines for task execution.	0.737	0.859
	EEP3	The company strives to provide monthly KPI reports to track profitability.	0.688	0.829
	EEP4	I have access to appropriate informational resources (e.g., IT facilities) for my work.	0.781	0.884
		Eigenvalue (λ): 2.895, Percentage of Variance Explained: 72.373%, Reliability (Cronbach’s Alpha): 0.870, KMO = 0.793—Bartlett’s Sphericity Test Significance 0.000		
Culture	CULT1	Our organization emphasizes planning, goal control, and achievement.	0.740	0.860
	CULT2	We value employees’ vision and growth to tackle new challenges.	0.742	0.861
	CULT3	Our organization focuses on participation/cooperation and member development.	0.692	0.832
	CULT4	Our organization values teamwork.	0.789	0.888
		Eigenvalue (λ): 2.962, Percentage of Variance Explained: 74.041%, Reliability (Cronbach’s Alpha): 0.882, KMO = 0.781—Bartlett’s Sphericity Test Significance 0.000		

The first PCA with a varimax rotation was conducted with all items and resulted in satisfactory factorial contributions, representational qualities, and reliabilities. The second exploratory factor analysis was performed on the culture scale using a PCA with a varimax rotation. The Kaiser Meyer Olkin measure (KMO = 0.781; *p* = 0.000) and the Bartlett’s test, indicating acceptable values, confirm the data factorization. The representational qualities and factorial contributions values are satisfactory as they are greater than 0.5. The culture scale exhibits a one-dimensional factorial structure. Indeed, the percentage of explained variance (equal to 74.041%) led to the selection of a single factor with an eigenvalue greater than 1 (=2.962). The observation of the Cronbach’s alpha coefficient for the “culture” variable measurement scale reveals that the scale also has good internal consistency.

### 4.2 Confirmatory factor analysis

#### 4.2.1 Measurement model for employee engagement and performance

In addition to the exploratory factor analysis, we conducted a confirmatory factor analysis (CFA) on the employee engagement and performance scale. The results show that the kurtosis and skewness indices are within acceptable norms, while Mardia’s index (14.422) far exceeds the minimum threshold of 3 (see Table 4).

**Table 4:** Multivariate normality results for the employee engagement and performance scale.

Variable	Min	Max	Skewness	Skewness C.R.	Kurtosis	Kurtosis C.R.
EEP1	1,000	5,000	−1.483	−13.227	1.965	8.759
EEP2	1,000	5,000	−1.106	−9.860	0.750	3.344
EEP3	1,000	5,000	−0.869	−7.748	−0.272	−1.212
EEP4	1,000	5,000	−1.250	−11.143	0.763	3.401
Multivariate					14.422	22.731

C.R. (Critical Ratio): absolute values > 1.96 indicate significant deviation from normality at *p* < 0.05. Multivariate: Mardia’s coefficient (14.422) exceeds the threshold of 3, and its critical ratio (22.731) confirms significant multivariate non-normality. Decimal commas are converted to periods. Negative skewness/kurtosis values indicate left-skewed or lighter-tailed distributions, respectively. This table highlights pronounced non-normality in EEP variables, necessitating robust statistical methods (e.g., MLR or bootstrapping) for valid CFA estimation. The Chi-square probability value (*p* = 0.006) was obtained using the maximum likelihood (ML) method.

The measurement model for employee engagement and performance demonstrates generally satisfactory fit indices. However, the normed Chi-square (χ^2^/df) is approximately 1.017. This value is below the threshold of 5 recommended by Marsh and Hocevar (1985, cited in Akrout, 2010) and further below the stricter maximum limit of 3 set by marketing researchers such as Carmines and McIver (1981, cited in Akrout, 2010) (see Table 5).

**Table 5:** Improvement of fit indices for the employee engagement and performance model.

Indices	Standardized Chi-square	GFI	AGFI	RMR	RMSEA	NFI	CFI	TLI
Value	1.017	0.999	0.999	0.011	0.010	0.997	0.998	0.927

Normed Chi-square (χ^2^/df): 1.017 (well below the recommended threshold of <3). GFI = Goodness of Fit Index; AGFI = Adjusted Goodness of Fit Index. RMR = Root Mean Square Residual; RMSEA = Root Mean Square Error of Approximation. NFI = Normed Fit Index; CFI = Comparative Fit Index; TLI = Tucker-Lewis Index. All values indicate excellent model fit, with GFI, AGFI, NFI, and CFI nearing 1.0 (perfect fit).

#### 4.2.2 Validation of the measurement model of employee engagement and performance

Following the exploratory analysis, we assessed the reliability and convergent validity of the employee engagement and performance measurement scale. The results were highly satisfactory, with a Jöreskog’s Rho (Composite Reliability) of 0.794 and a Convergent Validity Rho (Average Variance Extracted, AVE) of 0.632 (see Table 6).

**Table 6:** Confirmatory factor analysis results for the employee engagement and performance scale.

Les items	Standardized factor loadings	SMC	Critical ratio C.R.	*p*-value
EEP1	0.863	0.034	8.982	0.000
EEP2	0.777	0.048	12.043	0.000
EEP3	0.791	0.033	11.681	0.000
EEP4	0.747	0.033	12.654	0.000
Rhô de Jöreskog = 0.794				
Convergent Validity (AVE) = 0.632				

SMC = Squared Multiple Correlations (proportion of variance explained by the factor). Critical Ratio (C.R.): values > 1.96 indicate significance at *p* < 0.05. Composite Reliability ≥ 0.7 and AVE ≥ 0.5 confirm strong reliability and convergent validity (Hair et al., 2010). Decimal commas converted to periods for international standards. This table validates the measurement model’s robustness, with all factor loadings significant (*p* < 0.001) and reliability/validity thresholds met.

#### 4.2.3 Confirmatory factor analysis of the change management practices scale

In addition to the exploratory factor analysis (EFA), we conducted a confirmatory factor analysis (CFA) on the change management practices scale. The results indicated that the skewness and kurtosis indices were within thresholds established by empirical studies. However, Mardia’s coefficient (*M* = 99.694) far exceeded the maximum recommended limit of 3, suggesting significant multivariate non-normality (see Table 7).

**Table 7:** Fit indices of the measurement model for change management practices.

Absolute indices	Chi-square = 150.507; ddl = 91; *p* = 0.000; GFI = 0.960; AGFI = 0.941; RMR = 0.064; RMSEA = 0.037
Incremental indices	NFI = 0.789; CFI = 0.900; TLI = 0.868
Parsimony indices	Normed Chi-square (χ^2^/df) = 1.654

#### 4.2.4 Validation of the measurement model for change management practices

The validation of the measurement model for change management practices involves confirming the confirmatory reliability and validity of each dimension of these practices. First, we calculated Jöreskog’s Rho (composite reliability) for each dimension. The Rho values exceeded the threshold of 0.6, ensuring strong internal consistency.

To assess convergent and discriminant validity for each dimension, we applied the approach of Fornell and Larcker (1981). The Table 8 demonstrates acceptable convergent validity Rho (Average Variance Extracted, AVE) values, all surpassing the 0.5 threshold advocated by Roussel et al. (2002).

**Table 8:** Reliability and convergent validity of change management practices.

Variables	Jöreskog’s Rho	Rhône of convergent validity
Communication (COMM)	0.718	0.611
Employee Participation in Decision Making (EPDM)	0.695	0.590
Training (TRAIN)	0.754	0.514
Leadership (LEAD)	0.794	0.620

Discriminant validity has been verified; we propose to summarize the results in Table 9. Note that the diagonals of the table represent the extracted variance values that are greater than the squares of the correlations between the dimensions.

**Table 9:** Discriminant validity of change management practices.

Variables	Communication (COMM)	Employee (EPDM)	Training (TRAIN)	Leadership (LEAD)
Communication (COMM)	0.611			
Employee Participation in Decision Making (EPDM)	0.290	0.590		
Training (TRAIN)	0.514	0.108	0.514	
Leadership (LEAD)	0.320	0.064	0.152	0.620

#### 4.2.5 Validation of the culture measurement model

Furthermore, the reliability and convergent validity of the culture measurement scale are very satisfactory, with values of 0.702 for Jöreskog’s Rhô and 0.636 for convergent validity Rhô (see Table 10).

**Table 10:** Results of the confirmatory factor analysis conducted on the culture scale.

Items	Standardized factorial contributions	Squared multiple correlation (SMC)	C.R	*p*
CULT1	0.721	0.053	5.705	0.000
CULT2	0.717	0.050	3.832	0.000
CULT3	0.740	0.036	−3.754	0.000
CULT4	0.663	0.042	3.809	0.000
Jöreskog’s Rhô = 0.702				
Convergent validity Rhô = 0.636				

#### 4.2.6 Confirmatory analysis of the global measurement model

The examination of the Kurtosis and Skewness indices reveals that they are within normal ranges, while the Mardia index, which is approximately 205.501, significantly exceeds the minimum threshold of 3. To address this issue of deviation from normality, we employed the Bollen-Stine Bootstrap method (*N* = 2000), which assesses the effect of the violation of the normality assumption by providing a corrected probability value related to the Chi-square (Akrout, 2010).

#### 4.2.7 Fit indices of the global measurement model

The corrected Chi-square probability value was compared to that obtained by the maximum likelihood method without correction, and no significant difference was observed. The overall measurement model remains stable before and after Bootstrap, confirming the data stability. The fit indices have fairly satisfactory values. However, the GFI, AGFI, and NFI values are below 0.9. This can be explained by the complexity of our model. Nonetheless, the standardized Chi-square has a low value of 1.304. The RMR and RMSEA values are within the normal ranges (see Table 11).

**Table 11:** Fit indices of the global measurement model.

Absolute fit indices	Chi-square = 285.548; df = 219; *p* = 0.002; GFI = 0.950; AGFI = 0.932; RMR = 0.068; RMSEA = 0.025
Incremental fit indices	NFI = 0.973; CFI = 0.932; TLI = 0.915
Parsimonious fit indices	Standardized Chi-square = 1.304

We have verified the reliability and validity of the overall measurement model. Firstly, we calculated Jöreskog’s Rhô values, which indicate values higher than the threshold of 0.6 set by Bagozzi and Yi (1988, cited in Akrout, 2010).

Secondly, to calculate convergent and discriminant validity, we relied on the approach of Fornell and Larcker (1981). The Tables 12, 13 show acceptable values of the convergent validity Rhô index, which are higher than the threshold of 0.5 recommended by Roussel et al. (2002).

**Table 12:** Reliability and convergent validity of variables in the global measurement model.

Variables	Jöreskog’s Rhô	Convergent Validity Rhô
Communication (COMM)	0.718	0.611
Employee Participation in Decision-Making (EPD)	0.695	0.590
Training (TRAIN)	0.754	0.514
Leadership (LEAD)	0.794	0.620
Employee Engagement and Performance (EEP)	0.794	0.634
Culture (CULT)	0.702	0.636

**Table 13:** Discriminant validity of the global measurement model.

Variables	Communication (COMM)	Employee participation in decision-making (EPDM)	Training (TRAIN)	Leadership (LEAD)	Employee engagement and performance (EEP)	Culture (CULT)
Communication (COMM)	**0.611**					
Employee Participation in Decision-Making (EPDM)	0.290	**0.590**				
Training (TRAIN)	0.514	0.108	**0.514**			
Leadership (LEAD)	0.320	0.064	0.152	**0.620**		
Employee Engagement and Performance (EEP)	0.273	0.260	0.146	0.242	**0.634**	
Culture (CULT)	0.226	0.157	0.061	0.188	0.233	**0.636**

The bold numbers are located on the diagonal of the table, and they represent the square root of the Average Variance Extracted (√AVE) for each construct. √AVE (bold numbers) shows how much of the variance in the indicators (questions/items) of a latent construct is explained by that construct.

## 5 Validation of the structural model

The structural model of our research (see Appendix) exhibits satisfactory fit indices. The GFI, AGFI, TLI, and NFI indices are slightly below 0.9 but above 0.8. The RMR and RMSEA indices have low values. Lastly, the model meets the parsimony conditions with a standardized Chi-square value of 2.266 (see Table 14).

**Table 14:** Fit indices of the structural model.

Standardized Chi-square	GFI	AGFI	TLI	CFI	NFI	RMR	RMSEA
2.266	0.902	0.881	0.844	0.881	0.855	0.061	0.052

**Figure 3:**
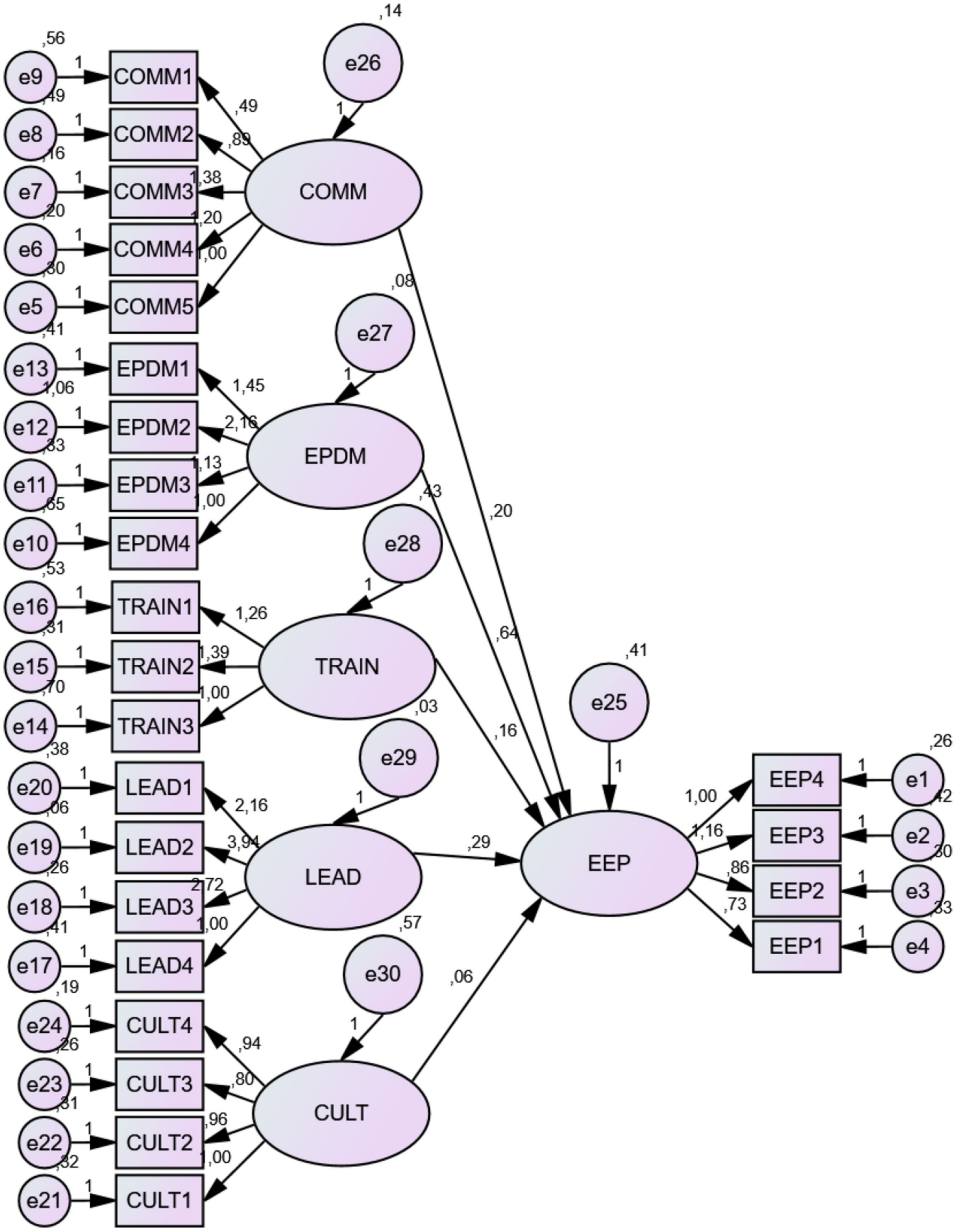
The causal model.

We used the Bootstrap procedure (*N* = 250) to verify the robustness of our model and address the issue of data non-normality (the Mardia index is above 3). The goal was to ensure that the difference between the parameter values estimated by the ML method and those from the bootstrapped samples is not significant (see Figure 3).

The structural model figure shows, first and foremost, that employee engagement and performance depend positively on change management practices and culture.

## 6 Results of direct relationship hypotheses by the method of structural equations

The different causality hypotheses between the variables are tested using the structural equations method in the software Amos version 23. The standardized regression coefficients (*γ*) and their level of significance are presented in the table below. Their interpretation would help to confirm or refute the hypotheses. We propose to present in the table below the results of the causal links between change management practices (Communication, Employee Participation in Decision-Making, Training, and Leadership), culture, and employee engagement and performance (see Table 15).

**Table 15:** Results of direct relationship hypotheses.

Variables	Coefficients	S. E.	C. R.	P	Conclusions
COMM→EEP	0.196	0.135	2.768	0.006	H.1 is validated
EPDM→EEP	0.641	0.230	2.781	0.005	H.1.1 is validated
TRAIN→EEP	0.159	0.063	2.512	0.012	H.1.2 is validated
LEAD→EEP	0.286	0.247	2.177	0.029	H.1.3 is validated
CULT→EEP	0.059	0.055	2.224	0.026	H.2 is validated

The results of the Structural Equation Modeling (SEM) indicate that all proposed hypotheses were statistically significant (*p* < 0.05), supporting the relationships between change management practices, cultural factors, and employee engagement and performance.

Specifically, culture (CULT → EEP) yielded a standardized coefficient (*β*) of 0.059, with a critical ratio (CR) of 2.224 and *p* = 0.026, thereby validating H2. Although statistically significant, the effect size is notably small, suggesting that cultural factors exert a relatively weak direct influence on employee engagement and performance compared to other change management dimensions such as participation (β = 0.641) or leadership (β = 0.286).

## 7 Discussion

We can conclude through the results that the first hypothesis (H1) of our research, which states that communication has a positive impact on employee engagement and performance, has been validated. Indeed, the structural link is significant at the 1% level (t = 2.768; *p* = 0.006). The regression coefficient between the two variables has a value of 0.196.

The first sub-hypothesis (H1.1) of our research, which states that employee participation in decision-making has a positive impact on employee engagement and performance, has been validated. Indeed, the structural link is significant at the 1% level (t = 2.781; *p* = 0.005). The regression coefficient between the two variables has a value of 0.641.

The second sub-hypothesis (H1.2) of our research, which states that training has a positive impact on employee engagement and performance, has been validated. Indeed, the structural link is significant at the 5% level (t = 2.512; *p* = 0.012). The regression coefficient between the two variables has a value of 0.159.

The third sub-hypothesis (H1.3) of our research, which states that leadership has a positive impact on employee engagement and performance, has been validated. Indeed, the structural link is significant at the 5% level (t = 2.177; *p* = 0.029). The regression coefficient between the two variables has a value of 0.286.

The second hypothesis (H2) of our research, which states that the culture of Saudi Arabia has a positive impact on employee engagement and performance, has been validated. Indeed, the structural link is significant at the 5% level (t = 2.224; *p* = 0.026). The regression coefficient between the two variables has a value of 0.059. Its effect size was very modest. This suggests that culture may not directly drive employee engagement and performance, but instead plays a background or moderating role in shaping how change initiatives are perceived and implemented. In Saudi Arabia’s high power-distance and collectivist culture, change may be accepted more due to authority and group conformity rather than personal belief in the change itself (Hofstede, 2001; House et al., 2004). This finding underscores the need for culturally sensitive leadership development and suggests that overt change strategies may need to be paired with deeper cultural understanding to achieve sustained impact.

Change management involves facilitating effortless participation in change while overcoming resistance and shock. Humans often prefer familiarity and convenience, hindering their ability to adapt to change.

The current study’s findings illuminate how Saudi Arabian firms’ cultures and change management strategies affect workers’ participation and attitudes. It implies that problems like traditionalism and religious views can be lessened with efficient tactics, alignment with cultural norms, and good communication. To encourage creativity and adaptation, the study suggests funding leadership development programs. It also suggests encouraging inclusivity and teamwork to propel practical transformation projects.

The study’s findings partially align with prior research conducted in Saudi Arabia and similar socio-cultural contexts. Al-Haddad and Kotnour (2015) exposes a strong correlation between change management practices and cultural factors in Saudi Arabian organizations, emphasizing the importance of effective strategies in influencing employee attitudes and engagement. However, our results challenge some assumptions. While culture was hypothesized to positively influence engagement and performance, the effect size was very small (*β* = 0.059), suggesting that culture may not operate as a direct driver, but rather as a background condition or moderator that shapes how other interventions are received.

Radu et al. (2023) suggested that a positive organizational culture fosters employee commitment, collaboration, and adaptability, ultimately enhancing attitudes and engagement levels. Almalki et al. (2021) highlighted the importance of integrating effective change management strategies with cultural considerations in Saudia Arabia organizations, ensuring sustainable employee engagement and a conducive environment for change initiatives. The study by Shash et al. (2020) stated that organizational change in contractors of Saudia Arabia is influenced by factors such as competition, new laws, growth, and economic crises, necessitating top management support and professional recruitment. Alsubaie (2022) suggested that Saudi Arabia’s business environment has evolved with the country’s World Trade Organization membership, women’s workforce, and Vision 2030 initiative. However, traditionalism and religious beliefs hinder change management. Effective communication, involving staff, and considering culture are key challenges. A Saudi Arabian study found that 70% of nursing professionals positively embraced the transition from paper-based to computer-based patient record systems, with nurse managers playing a crucial role in their support (Brand, 2013). Sabir et al. (2022) found a significant positive correlation between perceived organizational support and employee performance from Saudi Arabian IT firms, with affective commitment partially mediating this relationship, indicating crucial job completion. The study by Ali et al. (2020) explores the impact of entrepreneurial orientation, market orientation, total quality management, and organizational performance on Saudi Arabia’s SMEs, revealing that these factors enhance performance, job creation, and economic growth. Their study found a positive relationship between change-oriented leadership and university performance in Saudi universities, with organizational innovation facilitating this relationship. Executive leaders should focus on work design strategies (AlAnazi et al., 2022). Also, Skiba (2021) found that employee involvement during mergers in a free market economy reveals discrepancies in perceptions. It suggests cyclic engagement surveys can identify errors and increase involvement, aligning employee attitudes with organizational goals and managers’ expectations.

The practical implications presented will equip readers with the knowledge needed to navigate change effectively in this context. By examining real-world cases like e-government initiatives and urban planning, the study adapts global frameworks to local contexts. The article provides unique insights into how leadership fosters change readiness by empowering stakeholders and aligning them with the national vision, contributing original perspectives on driving organizational transformation in Saudi Arabia. These practical implications will equip the reader with the necessary knowledge to navigate change in the Saudi industrial sector.

With in-depth analysis and contextualizing of the findings within the Saudi Arabian industrial sector, the paper could bridge the gap between theory and practice more effectively. For research, the study contributes to the growing body of knowledge on how change management practices intersect with cultural factors, particularly within the unique socio-economic context of Saudi Arabia.

## 8 Conclusion

Throughout this article, we have highlighted the important role played by change management practices, including communication, employee participation in decision-making, training, leadership, and the culture of Saudi Arabia in employee engagement and performance. According to the results of the empirical study we conducted, it has been found that change management practices, namely communication, employee participation in decision-making, training, leadership, and the culture of Saudi Arabia play significant roles in improving employee engagement and performance. The analysis of the relationship between the dimensions of change management practices (communication, employee participation in decision-making, training, and leadership), and employee engagement and performance reveals that all dimensions of change management practices have positive and significant impacts on employee engagement and performance. Thus, for the relationship between the culture of Saudi Arabia and employee engagement and performance, the empirical results reveal a positive and significant impact.

This study contributes to the existing body of knowledge on change management in the Saudi organization context, providing valuable information for academic staff and leaders. Therefore, the authors recommended that organizations in Saudia Arabia should invest in leadership development programs to equip leaders with the necessary skills, competencies, and mindset for successful change initiatives, fostering a culture of innovation and adaptability. Saudi Arabian organizations should promote inclusivity and collaboration by fostering cross-cultural understanding, openness, respect, and appreciation for diverse perspectives, beliefs, and values and driving successful change initiatives. In addition, Leaders must recognize that while communication and leadership behaviors do influence employee attitudes, their effect sizes are modest, indicating that these actions alone are not transformative. Leaders should pair strategic communication with visible, consistent behaviors that reinforce change messages.

Furthermore, in terms of societal impact, the research may influence public attitudes towards organizational change and contribute to improving quality of life by promoting better work environments and job satisfaction in the context of national transformation efforts. However, to achieve a fuller understanding of these implications, a more detailed presentation of the survey methods and results is necessary, which would also enhance the discussion and provide greater clarity for applying these findings in real-world settings.

Practically, the findings suggest that Saudi firms must go beyond formal training and communication to foster change readiness. This includes building inclusive decision-making structures, addressing deep-seated resistance through cultural reframing, and empowering leaders to act as change agents aligned with national strategies like Vision 2030. Lessons from e-government projects and sustainable urban development illustrate the importance of tailoring change approaches to local contexts.

For HR professionals play a key role in shaping the employee experience during change. The strong effect of employee participation on engagement and performance (*β* = 0.641) highlights the need to move beyond traditional top-down communication. HR departments should Incorporate cultural awareness into training programs to ensure that leadership development aligns with local norms and values.

Given the national importance of organizational transformation under Vision 2030, policymakers can use these findings to support initiatives that institutionalize employee participation in both public and private sectors, including reforms to labor policies or corporate governance guidelines.

### 8.1 Limitations of the study

The study’s limitations included the self-reported data and considering broader variables—the study’s cross-sectional nature and the focus on selected private sectors in Saudi Arabia.

We analyzed the relationship between change management practices and cultural factors and made theoretical considerations on the direction in which organizational change should proceed through the analysis results. However, this study has a limitation in that it failed to discuss the procedural aspects of change management within the organizational culture, which may limit generalizability to other contexts. However, efforts were made to mitigate these limitations through rigorous sampling techniques and data analysis methods.

The Contextual saturation: In homogeneous environments like a single-country setting (Saudi Arabia), variability in responses may be naturally constrained, which can limit the observable strength of relationships.

Despite these limitations, small effect sizes should not be dismissed outright. In organizational research, even small effects can accumulate over time or scale meaningfully across a large number of firms.

### 8.2 Future research

Future studies should adopt short-term priorities such as improving cultural measurement tools, conducting moderation/mediation analyses, and exploring sector-specific differences. Long-term priorities include longitudinal and multi-level designs to track change over time and across organizational levels. Cross-cultural comparisons and mixed-methods approaches can further deepen theoretical and contextual insights.

## Data Availability

The original contributions presented in the study are included in the article/Supplementary material, further inquiries can be directed to the corresponding author.
