# Supplementary material for: Navigating change: the role of change management strategies and cultural factors in Saudi Arabian organizations
Source: Front Psychol. 2025 Sep 1;16:1551902. doi: 10.3389/fpsyg.2025.1551902 (PMC12434401; doi:10.3389/fpsyg.2025.1551902)
Supplement: Supplementary file 1 [file Table_1.docx]

**Appendix 1: Table S1: Exploratory Factor Analysis Results for the Change Management Practices Scale**

| **Items** | | Representation Qualities | Factorial Contributions | | | | | | |
| --- | --- | --- | --- | --- | --- | --- | --- | --- | --- |
|  |  |  | COMM | | EPDM | | Training | | **Leadership** |
| **COMM 1** | The company's strategy is clearly communicated at all hierarchical levels | 0,529 | 0,636 | |  | |  | |  |
| **COMM 2** | Information about the company and its action plans is disseminated at all levels in terms understandable to all | 0,542 | 0,689 | |  | |  | |  |
| **COMM 3** | The objectives set for individuals and teams are consistent with the company's strategy | 0,759 | 0,832 | |  | |  | |  |
| **COMM 4** | Employees are informed about upcoming transformations and their progress | 0,690 | 0,774 | |  | |  | |  |
| **COMM 5** | The values promoted by my company are clear and widely communicated | 0,635 | 0,704 | |  | |  | |  |
| **EPDM 1** | Our organization takes into account the opinions of all employees in decision-making | 0,566 |  | | 0,620 | |  | |  |
| **EPDM2** | We can ask for clarification or additional information about our decision-making process | 0,652 |  | | 0,793 | |  | |  |
| **EPDM 3** | In our institution, change generally has a positive effect | 0,608 |  | | 0,592 | |  | |  |
| **EPDM 4** | Employees can object to decisions made in our institution. | 0,569 |  | | 0,626 | |  | |  |
| **TRAIN 1** | I am constantly developing to improve my performance capability. | 0,724 |  | |  | | 0,844 | |  |
| **TRAIN 2** | I can have appropriate training/capacity development activities if I need to do my job. | 0,786 |  | |  | | 0,880 | |  |
| **TRAIN 3** | Recent training and capacity building activities have helped me in my professional performance. | 0,646 |  | |  | | 0,784 | |  |
| **LEAD1** | My boss provides me with a solid vision for the future. | 0,671 |  | |  | |  | | 0,764 |
| **LEAD2** | My boss motivates me to work hard. | 0,827 |  | |  | |  | | 0,870 |
| **LEAD3** | My boss encourages me to work from a new perspective. | 0,791 |  | |  | |  | | 0,848 |
| **LEAD4** | Leaders successfully engage in the change management process. | 0,573 |  | |  | |  | | 0,681 |
| **Eigenvalue (λ)** | | | | 5,694 | | 2,070 | | 1,376 | 1,228 |
| **Reliability (Cronbach's Alpha)** | | | | 0,827 | | 0,681 | | 0,800 | 0,869 |
| **Percentage of Explained Variance** | | | | 19,793% | | 19,085% | | 13,636% | 12,283% |
| **KMO** = 0,873 | | | | | | | | | |
| **Significance of Bartlett's Sphericity Test** = 0,000 | | | | | | | | | |

Appendix 2: Table S2. Assessment of normality

| Variable | min | Max | skew | c.r. | kurtosis | c.r. |
| --- | --- | --- | --- | --- | --- | --- |
| CULT4 | 1,000 | 5,000 | -1,277 | -11,382 | 1,354 | 6,035 |
| CULT3 | 1,000 | 5,000 | -1,449 | -12,920 | 1,836 | 8,184 |
| CULT2 | 1,000 | 5,000 | -1,146 | -10,215 | ,751 | 3,348 |
| CULT1 | 1,000 | 5,000 | -1,106 | -9,863 | ,556 | 2,480 |
| LEAD1 | 1,000 | 5,000 | -1,685 | -15,028 | 2,733 | 12,185 |
| LEAD2 | 1,000 | 5,000 | -1,567 | -13,972 | 1,962 | 8,746 |
| LEAD3 | 1,000 | 5,000 | -1,657 | -14,774 | 2,376 | 10,591 |
| LEAD4 | 1,000 | 5,000 | -1,406 | -12,534 | 1,601 | 7,138 |
| TRAIN1 | 1,000 | 5,000 | -,350 | -3,119 | -,714 | -3,184 |
| TRAIN2 | 1,000 | 5,000 | -,424 | -3,782 | -,590 | -2,629 |
| TRAIN3 | 1,000 | 5,000 | -,529 | -4,716 | -,497 | -2,214 |
| EIE1 | 1,000 | 5,000 | -1,408 | -12,558 | 1,535 | 6,845 |
| EIE2 | 1,000 | 5,000 | -,019 | -,173 | -1,158 | -5,163 |
| EIE3 | 1,000 | 5,000 | -1,737 | -15,491 | 2,850 | 12,708 |
| EIE4 | 1,000 | 5,000 | -1,494 | -13,319 | 1,825 | 8,136 |
| COMM1 | 1,000 | 5,000 | -1,313 | -11,709 | 1,560 | 6,956 |
| COMM2 | 1,000 | 5,000 | -,726 | -6,471 | -,024 | -,108 |
| COMM3 | 1,000 | 5,000 | -1,220 | -10,878 | 1,567 | 6,985 |
| COMM4 | 1,000 | 5,000 | -1,326 | -11,820 | 1,962 | 8,746 |
| COMM5 | 1,000 | 5,000 | -1,530 | -13,638 | 2,540 | 11,323 |
| EEP1 | 1,000 | 5,000 | -1,483 | -13,227 | 1,965 | 8,759 |
| EEP2 | 1,000 | 5,000 | -1,106 | -9,860 | ,750 | 3,344 |
| EEP3 | 1,000 | 5,000 | -,869 | -7,748 | -,272 | -1,212 |
| EEP4 | 1,000 | 5,000 | -1,250 | -11,143 | ,763 | 3,401 |
| Multivariate |  |  |  |  | 205,501 | 63,524 |
